# Pediatric Rheumatology Collaborative Study Group – over four decades of pivotal clinical drug research in pediatric rheumatology

**DOI:** 10.1186/s12969-018-0261-x

**Published:** 2018-07-11

**Authors:** Hermine I. Brunner, Lisa G. Rider, Daniel J. Kingsbury, Dominic Co, Rayfel Schneider, Ellen Goldmuntz, Karen B. Onel, Edward H. Giannini, Daniel J. Lovell, for the PRCSG Advisory Council, Suzette Peng, Sarah Yim

**Affiliations:** 1 Division of Rheumatology, Cincinnati Children’s Hospital Medical Center & Cincinnati Children’s Research Foundation, MLC 4010, Cincinnati, OH 45229 USA; 2 Environmental Autoimmunity Group, Clinical Research Branch, National Institute of Environmental Health Sciences, National Institutes of Health, Bethesda, MD USA; 3 Randall Children’s Hospital at Legacy Emanuel, Portland, OR USA; 4 University of Wisconsin School of Medicine and Public Health, Madison, WI USA; 5 The Hospital for Sick Children, University of Toronto, Toronto, Canada; 6 Division of Allergy, Immunology, and Transplantation, Rheumatologic Autoimmune Diseases Section, National Institutes of Health, Bethesda, MD USA; 7 Hospital for Special Surgery, Weill Cornell Medicine, New York, NY USA

**Keywords:** Clinical trials, JIA, PRCSG, Drug approvals, Pediatrics, Networks

## Abstract

**Importance:**

Specialized research networks are essential to achieve drug approvals for rare pediatric diseases. Such networks help realize the potential of global legislation enacted upon the recognition that most children are treated with drugs whose most beneficial dose and regimen have not been established in pediatric patients. The Pediatric Rheumatology Collaborative Study Group (PRCSG) is a North American clinical trials network that is specialized in the performance of clinical trials of new therapies for pediatric populations with rheumatic diseases. This review provides an overview of the strategies employed by this research network to achieve drug and biologic approvals for children with pediatric rheumatic diseases, particularly juvenile idiopathic arthritis.

**Observations:**

Clinical trial conduct in rare pediatric diseases has required global recruitment. Supported or led by the PRCSG, highly responsive, validated, composite measures have been established to assess drug efficacy. For pediatric orphan diseases with high disease burdens, specialized investigative sites and study designs are needed to complete adequately powered trials at the high standard necessary to enable drug labeling by regulatory agencies. Novel trial designs have been utilized for more efficient testing of innovative drug candidates. All these have been developed or co-developed by the PRCSG research network.

**Conclusions and relevance:**

Specialized research networks in pediatric rheumatology, such as the PRCSG, have changed the landscape of available therapies and improved overall disease outcomes for children with pediatric rheumatic diseases.

## Background

There remains a profound unmet medical need for the effective and safe treatment of pediatric rheumatic diseases. As these diseases are rare, highly specialized and experienced research networks are best suited to support therapeutic trials of new drug compounds. The Pediatric Rheumatology Collaborative Study Group (PRCSG) has an over 40-year long track record of successfully supporting the design, conduct, analysis, and publication of such therapeutic trials in close collaboration with industry sponsors. The PRCSG has intensely focused on ensuring that clinical trial designs have scientific rigor, are practical for enrollment by pediatric rheumatologists, and acceptable to families of children with pediatric rheumatic diseases. Studies conducted in collaboration with the PRCSG changed the landscape of pediatric rheumatology care over the past decades. The objective of this review is to highlight the history of the PRCSG, including approaches developed and used by the research network that made effective therapies available to children with pediatric rheumatic diseases.

## Food and Drug Administration regulations for drugs for children with pediatric rheumatic diseases

The Food and Drug Administration (FDA) is the federal agency charged with overseeing drug manufacturing, labeling, advertisement, and safety of medications and biological products [1] in the United States (U.S.). The Food, Drug, and Cosmetic Act (FD&C Act) requires that there is “substantial evidence,” resulting from “adequate and well controlled investigations” to demonstrate that a new drug “will have the effect it purports or is represented to have under the conditions of use prescribed, recommended, or suggested in the proposed labeling.” New drug and biologic applications are submitted to and reviewed by the FDA for potential approval for marketing in the U.S [2].

Activism by professional organizations, families, and networks, such as the PRCSG, resulted in legislation that markedly changed the landscape of drug therapy for children. In the U.S., medication labeling and drug studies are governed largely by two sections of the FD&C Act: section 505A pertains to The Best Pharmaceuticals for Children Act (BPCA) [3] and section 505B to the Pediatric Research Equity Act (PREA) [4], respectively. Together these two laws encourage and/or require drug companies to study their products in children [5]. PREA necessitates new drugs and biologic therapies to be studied in children, if there is a pediatric disease that is similar to a non-orphan disease occurring in adults, and it is likely that the new agent will be used in children. The FDA considers juvenile idiopathic arthritis (JIA) with polyarticular joint involvement the pediatric correlate of adult rheumatoid arthritis (RA) [6]. BPCA provides pharmaceutical manufacturers with 6 months of additional market exclusivity after the completion of pediatric drug studies that have been done at request of the FDA. There is no exclusion for orphan diseases, but biologic therapies are not covered. The FDA Safety and Innovation Act (FDASIA) made PREA and BPCA permanent, which was important because, prior to 2012, PREA and BPCA were merely regulations intended to be in place for a limited time. Almost all stakeholders involved, including leaders of the PRCSG, asserted that permanence of PREA and BPCA was an important aspect for future drug development for children. Together, these laws resulted in the labeling of more than 600 products for pediatric indications, 149 of which occurred since the passage of FDASIA [7]. A recent review by the European Medicines Agency (EMA) and FDA confirmed that the existence of pediatric research networks, such as the PRCSG, is of utmost importance to realize the potential of this new pediatric legislation [2, 8].

Integration of pediatric planning and exclusivity requests are now a part of regular new drug and biologic product development programs at pharmaceutical companies. Key documents to be developed include the initial Pediatric Study Plan (iPSP) for submission to and approval by the Center for Drug Evaluation and Research (CDER) or the Center for Biologics Evaluation and Research (CBER) at FDA [9].

Importantly, the EMA passed similar legislation concerning pediatric drug testing and approval. Like the iPSP, a Pediatric Investigational Plan (PIP) must be submitted to the Paediatric Committee (PDCO), EMA’s scientific committee that is responsible for activities concerning medicine testing in children. PDCO oversees the labeling of such medicines in the European Union. One difference between the U.S. and the European legislation is that PIP submission to PDCO takes place by the end of Phase I development in adults, while iPSP submission to FDA is expected to occur later around the end of Phase II development in adults.

It has been a long-term initiative of the FDA to facilitate international standardization in drug testing and drug approval (“harmonization”). Various FDA Centers coordinate regular conferences with colleagues in Europe, Japan, Canada, and Australia. The benefits of harmonization of drug and biologic DMARD testing in pediatric rheumatic diseases include a decrease of unnecessary exposure to placebo or potentially unsafe new medications. Harmonization efforts are especially evident in the context of pediatric drug trials. This is achieved by international agreement between the various regulatory agencies on the design and performance of only a single blinded controlled pediatric trial, rather than several studies.

## Research network focused on promoting access to new medications for pediatric rheumatic disease therapy

Founded in 1973, the PRCSG is a research network of nearly 90 academic clinical pediatric rheumatology centers. At present, there are over 180 pediatric rheumatology investigators in North America and Puerto Rico who are members of the PRCSG. The mission of the PRCSG (http://www.prcsg.org) is to foster, facilitate, and conduct high quality clinical research in the field of pediatric rheumatology. The PRCSG Advisory Council provides oversight of the network’s activities. Its members are established clinical investigators, a junior clinical investigator, and representatives of foundations, organizations, and government agencies pertinent to the mission of the network, such as the National Institutes of Health and FDA.

The impact of the PRCSG extends beyond North America. The PRCSG leadership was involved in the training of the leadership of the Paediatric Rheumatology International Trials Organisation (PRINTO; http://printo.it) [10], a research network that now includes centers in Europe, Latin America, Asia and Africa, and whose mission is aligned with that of the PRCSG. These two networks have been active collaborators for over 20 years, which ultimately supports licensure of new medicines in many countries throughout the world.

Studies in pediatric rheumatic diseases often require complex response measures; necessitate adjustment of background medications; and rules for early discontinuation of subjects for added safety. To ensure that study protocols are closely followed by the investigative sites, the PRCSG and PRINTO Coordinating Centers have developed standardized operating procedures, custom software, electronic data capture systems and databases, as well as data security and back-up systems. This robust infrastructure allows the Coordinating Centers to provide real-time feed-back to investigative sites throughout the world and across all time zones. Although focused on pharmaceutical supported trials, the PRCSG Coordinating Center also supports investigator-initiated studies [11, 12].

## The scientific collaboration treaty between the U. S. and Union of Soviet Socialist Republics supported early clinical trials in JIA

During the Cold War era, and as part of a Scientific Collaboration Treaty between the State Departments of the U.S. and the Union of Soviet Socialist Republics (USSR), the PRCSG conducted trials of popular traditional Disease Modifying Anti-Rheumatic Drugs (DMARDs) with pediatric rheumatologists in the U.S. and the USSR. The motivation behind the Scientific Collaboration Treaty was that the fostering of scientific collaboration between the two countries was hoped to promote collaborations in other realms. Dr. Earl Brewer, the founder and first chairman of the PRCSG, considered the Scientific Collaboration Treaty a unique opportunity to obtain federal funding for clinical trials in JIA.

Facilitated by the Scientific Collaboration Treaty, three large, pivotal Phase III randomized, double-blind, placebo-controlled trials of DMARDs were successfully performed through the collaborative efforts of pediatric rheumatologists in the U.S. and the USSR. The first study compared the efficacy of D-penicillamine and hydroxychloroquine to placebo in polyarticular JIA [13]. Despite promising results in open-label clinical studies, the results of the controlled study proved that neither D-penicillamine nor hydroxychloroquine were superior to placebo in JIA. Thus, this PRCSG study spared other children with JIA treatment with these ineffective medications. A second clinical trial compared the efficacy and safety of oral gold to that of placebo in polyarticular JIA. Despite earlier positive clinical trials of oral gold in RA, this PRCSG study supported that oral gold was only marginally superior to placebo and had considerable toxicity when used in JIA [14]. Taken together, this PRCSG trial again prevented JIA patients from treatment with minimally effective yet potentially toxic oral gold therapy.

The third trial was of methotrexate versus placebo in active polyarticular JIA [15]. Only oral methotrexate at 10 mg/m^2^ body surface area (BSA) once per week, but not the lower weekly dose of 5 mg/m^2^ BSA, proved to be superior to placebo in JIA. The results of this study were specifically used for the subsequent labeled indication of methotrexate in reducing signs and symptoms of polyarticular JIA.

Other trials performed by the PRCSG were of various nonsteroidal anti-inflammatory drugs (NSAIDs), which were formerly the cornerstone of JIA treatments [16–23]. These studies led to FDA approval of five NSAIDs for JIA (Table 1).

**Table 1:** Medications studied by the PRCSG Pediatric Rheumatology Collaborative Study Group for Juvenile Idiopathic Arthritis and other pediatric rheumatic diseases by route(s) of administration^a^

Medications studied but without approval/licensure by regulatory agencies	Medications studied with approval/licensure by regulatory agencies	Studies in Progress
Oral	Oral	Oral
D-penicillamine	Celecoxib	Baricitinib
Fenoprofen	Ibuprofen	Tofacitinib
Gold	Naproxen	Subcutaneous
Hydroxychloroquine	Rofecoxib^b^	*Certolizumab pegol*
Ketoprofen	Tolmetin	*Sarilumab*
Meclofenamate	Oral, subcutaneous or intramuscular	*Secukinumab*
Oxaprozin	Methotrexate	*Tocilizumab*
Pirprofen	Subcutaneous	Intravenous
Proquazone	*Adalimumab*	*Belimumab*^g^
Subcutaneous	*Canakinumab*^c^	*Golimumab*
*Anakinra*	*Etanercept*	*Rituximab*^h^
Intravenous	*Golimumab*^d^	
Intravenous immunoglobulin	Subcutaneous and/or intravenous	
*Infliximab*	*Abatacept*^e^	
*Rilonacept*	*Tocilizumab*^f^	

Biologic medications are printed in Italics

^b^Approval withdrawn by the U.S. Food and Drug Administration due to safety concerns in adults with rheumatoid arthritis

^c^Only for systemic juvenile idiopathic arthritis (JIA)

^d^Approved by European Medicines Agency only, but not by the U.S. Food and Drug Administration

^e^Both the intravenous and the subcutaneous form is approved for polyarticular JIA

^f^The intravenous forms are approved for systemic JIA and polyarticular JIA

^g^For systemic lupus erythematosus

^h^For antineutrophil cytoplasmic autoantibody (ANCA) associated vasculitis

## Clinical trials of biological DMARDs result in markedly improved prognosis of JIA with polyarticular joint involvement

The first biologic DMARD studied in JIA was etanercept, a fusion protein targeting tumor necrosis factor alpha (TNFα) [24]. The efficacy of etanercept in JIA was tested and established using the then novel randomized withdrawal design (RWD). This RWD study (*n* = 69) was fully enrolled within 3 months from sites in North America because many children with JIA had failed all available medications. When the first JIA patient was dosed with etanercept, only about 300 adult patients with RA had been treated with this drug. Etanercept remains the first and presently only biologic DMARD that achieved regulatory approval by the FDA using the “fast track” approach. Eventually, world-wide approval of etanercept for polyarticular JIA was achieved based on the results of this PRCSG trial. However, given its small sample size and short duration of follow-up, the FDA required the Sponsor to establish a large post-marketing Phase IV JIA registry to document the continued effectiveness, benefits on quality of life, and longer-term safety of etanercept in routine clinical use, including usage in younger children [25]. These studies resulted in important additions to the label for etanercept pertaining to medication safety events, and broadened the approval to children as young as 2 years. Subsequent to the etanercept study, there have been a series of clinical trials managed by the PRCSG and PRINTO, which resulted in FDA approval of a total of six biologic DMARDs for polyarticular JIA [24, 26–30].

In the last decade, PRCSG investigators also participated in clinical trials in systemic JIA (SJIA) [31–33]. The trials of canakinumab, an interleukin (IL)-1β monoclonal antibody, and the anti-IL-6 monoclonal antibody, tocilizumab, in SJIA led to the regulatory approval of both biologic DMARDs in North America and Europe [32, 33]. A list of all medications studied by the PRCSG, including those that are now licensed for JIA, is provided in Table 1.

With the advent of designer drugs that target pathways only relevant to some rare JIA subsets, the corresponding clinical trials in children constitute an even larger challenge. Examples are drugs that inhibit the IL-17 or IL-12/23 pathways which are expected to only benefit children with juvenile psoriatic arthritis and /or enthesitis-related JIA. The PRCSG has also been involved in the planning and conduct of the pediatric clinical trials of the anti-B lymphocyte stimulator monoclonal antibody, belimumab, in systemic lupus erythematosus (SLE), and the anti-CD20 monoclonal antibody, rituximab, in anti-neutrophil cytoplasmic antibody associated vasculitis.

## Global commitments and access to care

Since 2000, JIA medication trials generally involved pediatric rheumatology centers located all over the world, including in developing countries where access to affordable biologic DMARDs remains limited. The PRCSG and PRINTO have adopted the guiding principle that, irrespective of regulatory approval in the child’s country, study participants must be provided long-term access to the study agent, as long as it is deemed medically beneficial to the child. The mechanism by which medication is provided to former study participants varies country by country. Despite the added cost, to date, all pharmaceutical companies have agreed to this request of the PRCSG and PRINTO.

## Development of outcome and response measures for pediatric rheumatic diseases

The PRCSG published the first guidance papers concerning medication studies in JIA [34, 35]. Many of the recommendations from 1982 continue to be relevant: well validated outcome measures are deemed a *sine-qua-non* for the successful conduct of clinical trials. Members of the PRCSG Advisory Council have held the view that it is unethical and scientifically unacceptable to proceed with large scale trials, if validated response criteria [36] are unavailable. Often in collaboration with other pediatric rheumatic disease networks, the PRCSG has been an active participant in the development and validation of outcomes measures (see Table 2 for listing of developed measures). The most frequently used outcome measures and response criteria include those for JIA [37–41]. The PRCSG leadership has also been involved in the delineation of core outcome measures and response criteria for SLE [42–47] and juvenile dermatomyositis [44, 48, 49].

**Table 2:** Clinical trial outcome measures developed by the Pediatric Rheumatology Collaborative Study Group Leadership in collaboration with other pediatric rheumatology networks

PEDIATRIC RHEUMATIC DISEASE	Outcome measure	Reference
Juvenile Idiopathic Arthritis	Core set of Outcome Measures^a^	[35]
	Preliminary Definition of Improvement^a^	[36]
	Preliminary Flare Criteria^a^	[37]
	Provisional criteria for inactive disease & clinical remission^a^	[38]
	Macrophage Activation Syndrome^a^	[39]
Systemic Lupus Erythematosus	Disease activity	[40]
	Disease damage	[41]
	Core Set of Outcome Measures^a^	[42]
	Provisional Improvement Criteria^b^	[43]
	Preliminary Flare Criteria^b^	[44]
	Preliminary Inactive Disease Criteria^b^	[45]
Juvenile Dermatomyositis	Childhood Myositis Activity Assessment Scale^c^	[46]
	Core set measures of disease activity and damage^a^	[42]
	Provisional Criteria for Response to Therapy^a^	[47]

^a^Developed in collaboration with Pediatric Rheumatology International Trials Organization (PRINTO)

^b^Developed in collaboration with PRINTO and Childhood Arthritis and Rheumatology Research Alliance (CARRA)

^c^Developed in collaboration with Juvenile Dermatomyositis Disease Activity Collaborative Study Group (predecessor to International Myositis Assessment and Clinical Studies Group)

## Novel study designs to maximize study efficiency

**Fig. 1:**
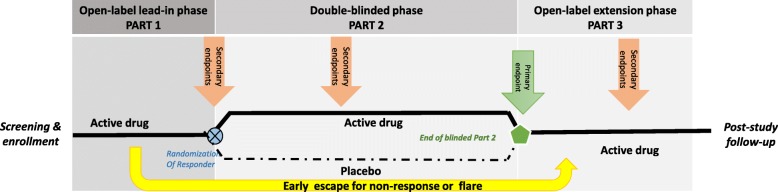
Basic design of Randomized Withdrawal Design. A randomized withdrawal design (RWD) study consists of three parts. During Part 1 and Part 3 all study participants receive open-label active study drug. Participants who show a clinically meaningful response to study drug by the end of Part 1 are randomized to the double-blinded placebo-controlled Part 2. Participants move from Part 2 to Part 3 if there is a flare event during Part 2 or upon completion of all Part 2 visits, whichever comes first. Patients for whom study drug may be beneficial, but who did not meet criteria for a meaningful improvement during Part 1, may be allowed to enter Part 3. The primary endpoint of a RWD trial is ‘the time to disease flare or the occurrence of a flare event during Part 2. The participant’s disease status at the end of Part 1 is used as the baseline to assess whether disease worsening (flare) has occurred during Part 2. Secondary RWD study endpoints can be measured throughout the duration of the entire RWD trial (Part 1 through 3) and include achievement of inactive disease, success in tapering certain background medications and change in patient reported outcomes

Pediatric rheumatic diseases are uncommon, and almost all affect fewer than 200,000 individuals in the US, which is compared to 1.3 million adult patients diagnosed with RA [50]. Pediatric rheumatic diseases are considered orphan or rare diseases as defined by the Orphan Drug Act [51]. Although JIA is the most common pediatric rheumatic disease, its prevalence is only 44.7 (95% CI 39.1-50.2) per 100,000 persons in the U.S. [52]. Thus, children with pediatric rheumatic diseases are a precious resource, and adequately powered clinical trials require an international enrollment strategy. Considering the burden of active pediatric rheumatic diseases, it is also critical for controlled studies to minimize placebo exposure while maximizing the collection of safety data. Therefore, the PRCSG has developed and used a randomized withdrawal design (RWD) for many of the controlled trials [24, 27, 29]. Beyond pediatric rheumatology, the RWD is now also used for the studies in, among others, gastrointestinal and neuropsychiatric diseases [53, 54]. As shown in Fig. 1, RWD trials provide active study drug to all study participants during the open label lead-in period (Part 1). Only subjects who have experienced a protocol-defined improvement in disease signs and symptoms will be randomized to enter the blinded placebo-controlled of a RWD trial (Part 2), while non-responders are either discontinued from the study at the end of Part 1 or allowed enter an open-label extension phase with access to active study drug (Part 3). All subjects who complete the blinded Part 2 can continue to receive active drug during Part 3. Study participants remain in the blinded treatment phase until the last visit in Part 2 or until he/she experiences a ‘disease flare’, i.e., a protocol-defined disease worsening, whichever comes first. Therefore, study participants remain in Part 2 only as long as they continue to demonstrate at least a similar level of disease control as at the beginning of Part 2, which is better than at the beginning of Part 1. The primary endpoint of a RWD trial is either the proportion of patients with ‘disease flare’ compared to the time of randomization, or the time to ‘disease flare’ in Part 2. Advantages of the RWD include a high degree of efficiency in assessing drug efficacy, requiring fewer patients to be enrolled in the trial; an individualized duration of exposure to placebo with an option to restart open-label active drug upon experiencing a ‘disease flare’. The disadvantages of the RWD trial are as follows: (1) there may exist a selection bias because only subjects who responded in the open lead-in phase are randomized into the double-blind phase, while children who failed to respond Part 1, and hence may have a lesser probability of favorable response to drug, are excluded from Part 2; (2) the primary outcome is not a direct comparison of response rates between treatment arms, but rather an indirect one (i.e., ‘disease flare’); (3) given that patients receiving placebo in the blinded portion are more likely to have a ‘disease flare’ and exit Part 2 early, there is often only a small blinded placebo safety exposure dataset. Despite these limitations, the RWD has functioned well in numerous JIA trials [24, 27–29, 33].

Use of traditional double-blind placebo-controlled parallel design may be considered in pediatric rheumatic diseases if (1) the new drug is fast-acting, hence the duration of placebo exposure is expected to be very short; (2) the new drug is not dosed continuously or has a very prolonged effect and therefore awaiting disease flare after therapy withdrawal would be impractical; or (3), there are safety concerns with sudden drug discontinuation of study drug in Part 2 of the RWD trials [32]. Whenever parallel designs are used, the PRCSG strongly advocates that there are liberal, early escape rules and an open label extension study. Such aspects of clinical development program ensure that patients with poor disease control have access to active drug early and over extended time periods.

In addition to placebo-controlled studies, the PRCSG uses open-label designs of studies for drug labeling purposes, provided the mechanism of action of a given drug is known to consistently result in improvement of a rheumatic disease in both children and adults, considering earlier controlled studies [55]. Open-label studies of “me-too” medications assume that the risk-benefit profile of a given drug is comparable to that of other drugs with a similar mechanism of action. The primary objective of such open-label studies in JIA is the identification of dosing regimens that result in drug exposures that have been found efficacious in RA. Regulatory agencies accepted an open-label study of certolizumab pegol in JIA (NCT01550003). This monoclonal antibody blocks TNFα-mediated inflammation in a similar manner to infliximab, adalimumab, and golimumab, i.e. biological DMARDs that have been found to be efficacious in controlled studies in RA and JIA. Other examples in JIA include intravenous golimumab (NCT02277444), and the study of subcutaneous abatacept.

Modification of the above-mentioned controlled and uncontrolled study designs include elements considered in adaptive trial designs. Examples are the reduction of sample size following planned interim analyses, the tapering of corticosteroids during the RWD trial of canakinumab in SJIA [33]; and protocolized discontinuation of methotrexate, NSAIDs, and even study drug, during the long-term phase of tocilizumab trial in SJIA [32].

Taken together, the PRCSG advocates study designs for new drugs that minimize placebo and background medication exposures. Nonetheless, any study by the PRCSG maintains sufficient scientific rigor needed for subsequent drug approval by regulatory agencies. Another prerequisite of PRCSG trials is that large-scale Phase III studies only proceed after the delineation of the dosing regimen that provides comparable drug exposure to those known to be efficacious in the related adult rheumatic disease. If the pathogenesis of the pediatric disease is distinct from the approved adult disease (e.g., RA and SJIA), dose-finding Phase II studies must be performed prior to the initiation of the Phase III trials used for achieving marketing approval [32]. Phase II dose finding studies were performed for both canakinumab and tocilizumab in SJIA [56, 57].

## Other activities of the PRCSG

Besides optimizing study designs, the PRCSG is actively engaged in the training of pediatric rheumatology investigators. This includes formal training and certification of quantitated reporting of the joint evaluation in children (Joint Assessor Certification) [58] and the training in the completion of other outcome measures for clinical trials. The PRCSG also provides special mentoring and training to investigators or pediatric rheumatology sites new to drug trials. In recognition of the changing landscape of medical care, the PRCSG now actively invites membership applications from nurse practitioners, physician assistants, and pediatric rheumatology trainees involved in the care of children with rheumatic diseases.

It is the intent of the PRCSG to facilitate translational research studies in pediatric rheumatic diseases. This has proven to be difficult as pharmaceutical firms are determined to minimize trial costs and logistic demands. Generally, the collection of biological samples during studies paid for by biopharmaceutical companies focus on the demands of regulatory agencies and the pharmaceutical sponsor in-house research portfolio. To make matters worse, there were no internationally-accepted standard protocols for the collection, shipping, processing, and storage of pediatric rheumatology biological samples until recently. This short-coming has been addressed by the international consortium UCAN (Understanding Childhood Arthritis Network) [36, 59]. The PRCSG interacts with UCAN in supporting translational research by pediatric rheumatology investigators that make use of samples and data from drug trials. A recent example is the gene profiling study in SJIA patients treated with canakinumab [60].

## Conclusions

**Fig. 2:**
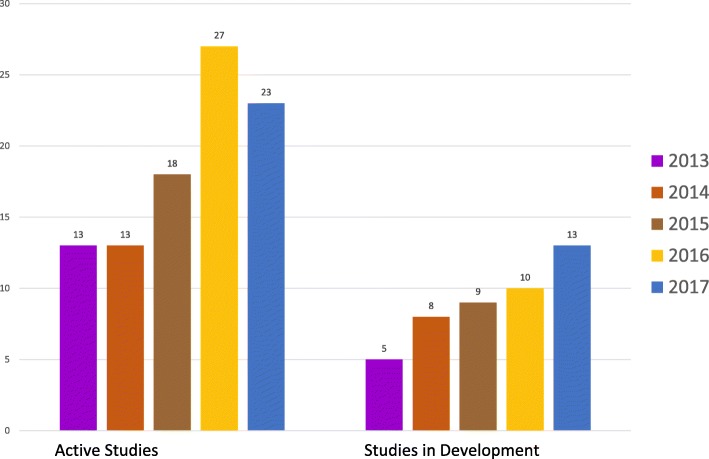
The PRCSG is a productive collaborative research network with focus on medication studies in pediatric rheumatic diseases. The PRCSG network has been continuously active with the number of studies active or in development markedly increasing since the passage of FDA Safety and Innovation Act (FDASIA) in 2012

Since the 1970’s, the PRCSG has contributed to the methodology of performing trials in pediatric rheumatic diseases. Closely collaborating with PRINTO and industry sponsors, 25 drugs and biologic agents have been studied by the network, resulting in 13 drug and biologic therapies achieving labeling for JIA by the FDA and/or EMA as of 2018. These drugs have dramatically improved the outcomes of children with polyarticular and systemic forms of JIA, leading to improved growth, quality of life, and possibly reduced radiographic joint erosions [61–65]. Upcoming clinical studies undertaken by the PRCSG (see Fig. 2) are expected to yield the licensure of additional drugs for children with various subsets of JIA and other pediatric rheumatic diseases.

## Abbreviations

BPCA: Best Pharmaceuticals for Children Act
BSA: Body surface area
CBER: Center for Biologics Evaluation and Research
CDER: Center for Drug Evaluation and Research
DMARDs: Disease Modifying Anti-Rheumatic Drugs
EMA: European Medicines Agency
FD&C Act: Food, Drug, and Cosmetic Act
FDA: Food and Drug Administration
FDASIA: FDA Safety and Innovation Act
IL: Interleukin
iPSP: Initial Pediatric Study Plan
JIA: Juvenile idiopathic arthritis
PDCO: Paediatric Committee
PIP: Paediatric Investigational Plan
PRCSG: Pediatric Rheumatology Collaborative Study Group
PREA: Pediatric Research Equity Act
PRINTO: Paediatric Rheumatology International Trial Organisation
RA: Rheumatoid arthritis
RWD: Randomized withdrawal design
U.S.: United States
UCAN: Understanding Childhood Arthritis Network
USSR: Union of Soviet Socialist Republics
